# Emerging MDR-*Mycobacterium avium* subsp. *avium* in house-reared domestic birds as the first report in Egypt

**DOI:** 10.1186/s12866-021-02287-y

**Published:** 2021-08-26

**Authors:** Abdelazeem M. Algammal, Hany R. Hashem, Amenah S. Al-otaibi, Khyreyah J. Alfifi, Esraa M. El-dawody, Eman Mahrous, Helal F. Hetta, Ali W. El-Kholy, Hazem Ramadan, Reham M. El-Tarabili

**Affiliations:** 1 Department of Bacteriology, Immunology and Mycology, Faculty of Veterinary Medicine, Suez Canal University, Ismailia, 41522 Egypt; 2 Department of Microbiology and Immunology, Faculty of Pharmacy, Fayoum University, Fayoum, 63514 Egypt; 3 Biology Department, College of Sciences, Tabuk University, Tabuk, 71491 Saudi Arabia; 4 Animal Health Research Institute, Dokki, Giza, 12618 Egypt; 5 Department of Medical Microbiology and Immunology, Faculty of Medicine, Assuit University, Assuit, 71515 Egypt; 6 Hygiene and Zoonoses Department, Faculty of Veterinary Medicine, Mansoura University, Mansoura, 35516 Egypt

**Keywords:** *M. avium subsp. avium*, House-reared domestic birds, prevalence, Antibiogram, MDR, antibiotic resistance-related genes

## Abstract

**Background:**

Avian tuberculosis is a chronic and zoonotic disease that affects a wide variety of birds, mammals, and humans. This study aimed to estimate the frequency of *Mycobacterium avium* subsp. *avium* in some domestic birds based on molecular diagnosis, antibiogram profile, and PCR-based detection of *inh*A, *rpo*B, *rps*L, and *otr*B antibiotic resistance-related genes.

**Methods:**

A total of 120 fecal samples were collected from small flocks of house-reared domestic birds at Ismailia Governorate, Egypt. The collected samples were processed and subjected to the bacteriological examination. The antimicrobial susceptibility testing of the recovered isolates was performed using the broth microdilution method for the detection of minimum inhibitory concentrations (MICs). The genetic detection of the *IS901*confirmatory gene, *inh*A*, rpo*B, *rps*L, and *otr*B genes was carried out using PCR.

**Results:**

The frequency of *M. avium* subsp. *avium* was 4.1% (5/120); 10% (4/40) in ducks, and 2.5% (1/10) in geese. The identification of the recovered isolates was confirmed using PCR, where all the tested isolates were positive for *IS901*confirmatory gene. The results of the broth microdilution method revealed that most of the recovered isolates exhibited multidrug resistance (MDR) to isoniazid, rifampicin, streptomycin, oxytetracycline, and doxycycline, and harbored the *inh*A*, rpo*B*, rps*L, and *otr*B genes.

**Conclusion:**

In brief, to the best of our knowledge this is the first report that emphasized the emergence of avian tuberculosis in house-reared domestic birds in Egypt. The emergence of MDR- *M. avium* subsp. *avium* is considered a public health threat. Emerging MDR-*M. avium* subsp. *avium* in domestic birds are commonly harbored the *IS901, inh*A*, rpo*B*, rps*L, and *otr*B genes. Azithromycin and clofazimine revealed a promising in-vitro antibacterial activity against *M. avium* subsp. *avium*.

## Background

Avian tuberculosis is a chronic, debilitating disease that affects a wide variety of birds including wild and domestic birds [1]. The disease is caused mainly by *Mycobacterium avium * subsp. *avium* serotypes 1, 2, and 3, and genotype *IS901* segment [2]. *Mycobacteria* are Gram-positive, aerobic, non-sporulated, non-motile, acid fast-bacilli. *Mycobacteria* are characterized by their lipid-rich cell wall which constitutes about 60–80% of their cell wall. *Mycobacteria* are opportunistic, intracellular pathogens that could withstand inside the macrophages and resist the host immune mechanism [3]. The bacilli of *M. avium* subsp*. avium* could resist most disinfectants, survive and multiply during adverse environmental conditions such as extreme temperature, low oxygen, and low pH [4].

*M. avium* subsp*. avium* transmitted between birds through the ingestion of contaminated food and water, inhalation of contaminated droplets, and from the adult birds to their young during the mouth feeding [5]. The infected birds with *M. avium* subsp. *avium* remain alive for a long time carrying the infection and subsequently shedding the pathogen in their dropping that results in the transmission of infection to other birds as well as humans [6, 7]. The infected birds may exhibit certain clinical signs such as diarrhea, emaciation, atrophy of breast muscle, and the development of tuberculous nodules in the last stage of the disease, especially in internal organs such as the intestine, liver, spleen, and lung [6].

Avian tuberculosis has public health importance, especially with immunocompromised persons that handle the infected birds or eat their insufficiently cooked meat [8, 9]. Most of the infected cases with *M. avium* subsp. *avium* are keeping domestic or pet birds in their home [10]. *M. avium* subsp. *avium* not only infects birds, but also could infect pet animals, pigs, and immunocompromised humans. Avian tuberculosis leads to high economic losses including; 1-High condemnation rates in poultry slaughterhouses, 2-Drop in egg production, 3-Weight loss and emaciation of infected birds, 4-Sudden death, and high mortality rates, and 5-Loss of endangered species of birds [11–13]. Avian tuberculosis is considered a serious problem in wild birds, especially the endangered species, so the valuable species should be checked regularly [14].

*M. avium* subsp*. avium* has a complex thick cell wall that is responsible for the intrinsic multidrug resistance and virulence [15, 16]. The diagnosis of *M. avium complex*, especially *M. avium* subsp. *avium* is difficult; as there are no specific clinical signs, it depends on;1-The culture technique that is recommended by OIE as the gold-standard technique, 2-The microscopical examination of acid-fast bacilli using Ziehl-Neelsen staining, and 3- PCR-based confirmatory diagnosis [10, 17, 18].

The multidrug resistance phenomena have increased globally. The emergence of multidrug-resistant bacterial pathogens from various origins was reported by several recent studies that reflect public health threats [19–26]. Treatment of avian tuberculosis is extremely difficult due to the development of antimicrobial resistance of *M. avium complex* to most antibiotics. The antimicrobial resistance is attributed to either intrinsic factors such as the waxy lipoid cell wall or mutation in some genes [27]. The most effective drug used in the treatment of avian tuberculosis is azithromycin [28]. Most types of *M. avium complex* have a weak response to the treatment due to the development of antibiotic resistance [27]. This is the first report concerning the emergence of MDR-*M. avium* subsp. *avium* in house-reared domestic birds in Egypt. This study aimed to estimate the frequency of *Mycobacterium avium* subsp. *avium* in some domestic birds based on molecular diagnosis, antibiogram profile, and PCR-based detection of *inh*A, *rpo*B, *rps*L, and *otr*B antibiotic resistance-related genes.

## Materials and methods

### Sampling

A total of 120 fecal samples from different domestic birds’ species (chicken (*n* = 40); 20 apparently healthy and 20 diseased birds, ducks (*n* = 40); 20 apparently healthy and 20 diseased birds, and geese (*n* = 40); 20 apparently healthy and 20 diseased birds) were collected from Private house-reared flocks from August 2018 to March 2019 from Ismailia Governorate, Egypt. The diseased birds were suffered from persistent diarrhea and emaciation together with a history of treatment failure with oxytetracycline. The collected samples were prepared according to Parashar et al. [29] and Payeur [30].

### Decontamination of fecal samples

The collected specimens were diced by sterile disposable surgical blades and then homogenized in a sterile porcelain mortar and pestle. The homogenized samples were suspended in 10 ml of PBS (Thermo Fisher Scientific, USA). The decontamination was performed as described by Sattar et al. [31]. Briefly, samples were inoculated in 0.9% Hexadecylpryridinium Chloride Monohydrate (HPC) (Sigma-Aldrich, USA), and then were incubated at 37 °C for 24 h. The mixture was centrifuged at 10 °C (3000 x*g*) for 15 min. The obtained pellets were re-suspended in 1 ml of sterile D.W., then were mixed (using vortex/ 500 rpm for 30 s.) with an equal volume of antibiotic suspension (vancomycin: 100 μg/ml, nalidixic acid: 100 μg/ml, and amphotericin: 50 μg/ml) (Oxoid, UK), followed by incubation at 37 °C for 24 h.

### Isolation and identification of *M. avium* subsp. *avium* from fecal samples

The processed fecal samples were streaked on Middlebrook 7H10 agar (Oxoid, UK); media was supplemented by a- 5 ml/l Glycerol (Oxoid, UK) and b-Middlebrook OADC Growth Supplement (Oxoid, UK). The inoculated plates were incubated at 37° ± 2 °C under microaerophilic conditions where a CO_2_ sachet (Oxoid, UK) is placed in a tightly closed anaerobic jar. The incubated plates were examined for bacterial growth at 2, 4, 6, 8, and 10 weeks post-incubation. The suspected colonies were identified according to their culture characters, morphological characters using Ziehl-Neelsen staining, and biochemically using niacin production, nitrate reduction, tween-80 hydrolysis, thermo-stable catalase at 68 °C, and arylsulfatase tests as described by Kubica [32]. Besides, the identification of the recovered isolates was confirmed by PCR-based detection of the *IS901* gene (the most conserved gene in *M. avium* subsp. *avium*) as described in Table 1.

**Table 1:** List of oligonucleotide sequences and cycling conditions that used in this study

Target genes	Oligonucleotide sequences (5′-3′)	Product size (bp)	Initial D	Cycling (35)			Final E
				D	A	E	
*M. avium* *IS901*	(F) 5′-GGATTGCTAACCACGTGGTG-3′	577	94 °C 5 min.	94 °C 30 s.	58 °C 40 s.	72 °C 45 s.	72 °C 10 min.
	(R) 5′- GCGAGTTGCTTGATGAGCG-3′						
*rpo*B	(F) 5′- TCAACATCCGTCCCGTCG-3′	347	94 °C 5 min.	94 °C 30 s.	60 °C 40 s.	72 °C 45 s.	72 °C 10 min.
	(R) 5′-GGCGGTCAGGTAGTGGAT-3′						
*rps*L	(F) 5′-ACCAGTTGCGACCCGTAGA-3′	592	94 °C 5 min.	94 °C 30 s.	56 °C 40 s.	72 °C 45 s.	72 °C 10 min.
	(R) 5′-CGCCTAACCGTAAGGAAGTGAA-3′						
*otr*B	(F) 5′-CCGACATCTACGGGCGCAAGC-3′	947	94 °C 5 min.	94 °C 30 s.	68 °C 40 s.	72 °C 1 min.	72 °C 10 min.
	(R) 5′-GGTGATGACGGTCTGGGACAG-3′						
*inh*A	(F) 5′ - TGGTCAGCTTCCTGGCTTCC-3′	810	94 °C 4 min	95 °C 1 min	55 °C 2 min	72 °C 2 min	72 °C 5 min
	(R) 5′ - GACCGTCATCCAGTTGTAG-3′						

*D* Denaturation, *A* Annealing, *E* Extension

### Antimicrobial susceptibility testing using broth microdilution method

The detection of minimal inhibitory concentrations (MICs) of the tested antimicrobial agents was carried out using the broth microdilution method according to the procedures of CLSI [33]. The test was performed using Middlebrook 7H10 broth (Oxoid, UK). The MIC for each antibiotic for each tested isolate was the mean of two repeated tests. *Mycobacterium avium* ATCC 700898 was used as a control strain. The MIC breakpoints of the tested antimicrobial agents were expressed as sensitive, intermediate, and resistant as described by the CLSI guidelines. The tested isolates were classified into Multidrug-resistant (MDR: the resistance to at least isoniazid and rifampicin), Extensively drug-resistant (XDR: resistant to isoniazid and rifampicin as well as to fluoroquinolones, and at least one of the second-line drugs such as kanamycin, amikacin, and capreomycin), and Pan-drug resistant (PDR: resistant to all antimicrobial agents listed) according to Prasanna and Niranjan [34].

### Molecular detection of *IS901*, *inh*A, *rpo*B*, rps*L, and *otr*B genes

PCR was used to detect the *IS901* gene to confirm the diagnosis of the recovered isolates as well as to investigate the presence of *inh*A, *rpo*B*, rps*L, and *otr*B genes in the recovered isolates. Extraction of DNA was performed using the QIAamp DNA Mini Kit (Qiagen, GmbH, Germany/ Catalogue No.51305). The PCR reaction performed in a “25- μl” reaction volume containing; “12.5 μl” of Emerald Amp Max PCR Master Mix (Takara, Japan), one μl of each primer of 20 pmol concentration, 4.5 μl of water, and 6 μl of DNA template. The oligonucleotide primers sequences (Metabion International AG, Germany) and their recycling conditions are illustrated in Table 1 [35–39]. Positive control strains (kindly provided by A.H.R.I, Egypt) and negative controls (DNA-free) were involved in each PCR run. Finally, the agar gel electrophoresis was carried out using 1.5% agarose stained with ethidium bromide 0.5 μg/ml (Fermentas, Germany). The gel was visualized by a gel documentation system (Thermo Fisher Scientific, Waltham, MA, USA).

### Statistical analyses

The statistical analysis of the obtained findings was carried out using Chi-square (SAS software, version 9.4, SAS Institute, Cary, NC, USA) (significance level; *P* < 0.05). The correlation analyses were performed between different tested antimicrobial agents as well as the antibiogram results and the presence of resistance genes using R software (version 4.0.2; https//www.r-project.org/).

## Results

### Phenotypic characteristics of the recovered *M. avium* subsp. *avium* from house-reared domestic birds

In the present study, the colonies of the recovered *M. avium* subsp*. avium* isolates were small, round, creamy color, and sticky on a Middlebrook 7H10 agar. The colonial growth was obtained within 2 weeks after incubation. The microscopical examination of the retrieved isolates (using Ziehl-Neelsen staining) revealed non-sporulated and non-motile acid-fast bacilli. The recovered isolates were positive for the arylsulfatase test, while were negative to the niacin production, nitrate reduction, thermo-stable catalase, oxidase, and tween − 80 hydrolysis tests (Table 2).

**Table 2:** Phenotypic characteristics of *M. avium* subsp*. avium*

Items	Phenotypic characteristics of ***M. avium*** subsp***. avium***					
**Colonies on Middlebrook 7H10 agar**	Small, round, creamy colored, and sticky colonies					
**Microscopical examination using Ziehl-Neelsen staining**	Non-sporulated, and non-motile acid fast bacilli					
**Biochemical characteristics**	**Test**	**Niacin production test**	**Nitrate reduction test**	**Tween-80 hydrolysis assay**	**Catalase test at 68 °C**	**Arylsulfatase Test**
	**Observation**	Clear liquid	No color change	No color change	No bubble was produced	Pink color produced within few seconds after adding sodium carbonate
	**Results**	-ve	-ve	-ve	-ve	+ve

### The frequency of *M. avium* subsp*. avium* in different species of examined domestic birds

**Fig. 1:**
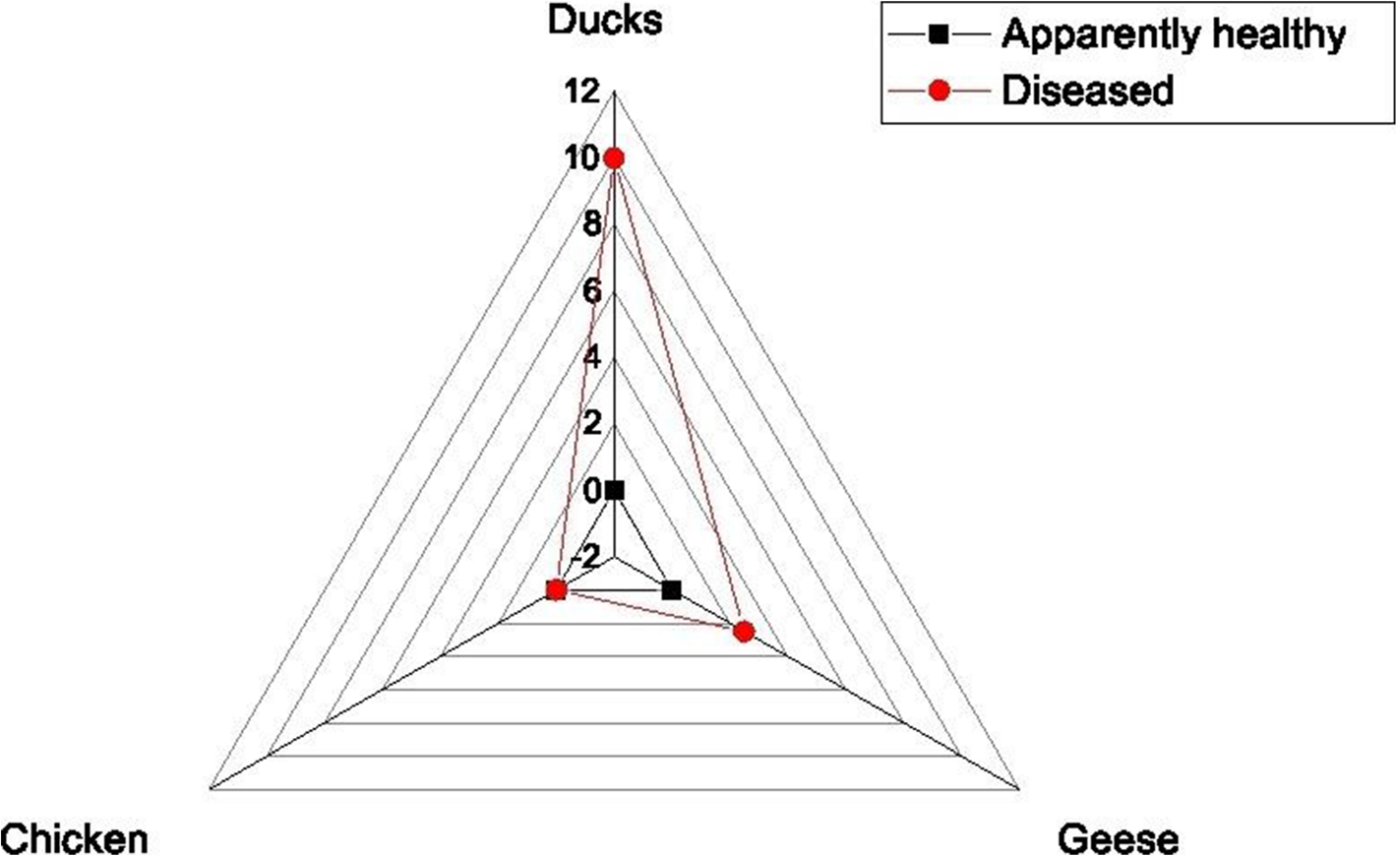
The frequency of *M. avium* subsp. *avium* among the examined domestic birds (Ducks, geese, and chicken)

The bacteriological examination revealed that the frequency of *M. avium* subsp*. avium* was 10% (4/40) in the examined ducks and 2.5% (1/40) in the examined geese. Moreover, the examined chicken samples were negative to *M. avium* subsp. *avium.* The total frequency of *M. avium* subsp. *avium* was 4.1% (5/120). All the retrieved isolates were originated from diseased birds suffering from persistent diarrhea and emaciation (Table 3 and Fig. 1). Statistically, there is a significant difference (*P* < 0.5; *P* = 0.008783) in the frequency of *M. avium* subsp*. avium* among examined samples of different bird species.

**Table 3:** The frequency of *M. avium* subsp*. avium* among different examined birds

Species of bird	No. of collected samples		No. of positive samples		Percentage of positive samples	
	Apparently healthy	Diseased	Apparently healthy	Diseased	Apparently healthy	Diseased
**Chicken**	20	20	0	0	0	0
**Ducks**	20	20	0	4	0	10 (4/40)
**Geese**	20	20	0	1	0	2.5 (1/40)
**Total**	120		5		4.1 (5/120)	
**Chi square** ***P*value**			15.4 0.008783

### Antibiogram of the recovered *M. avium* subsp*. avium* isolated from domestic birds

**Fig. 2:**
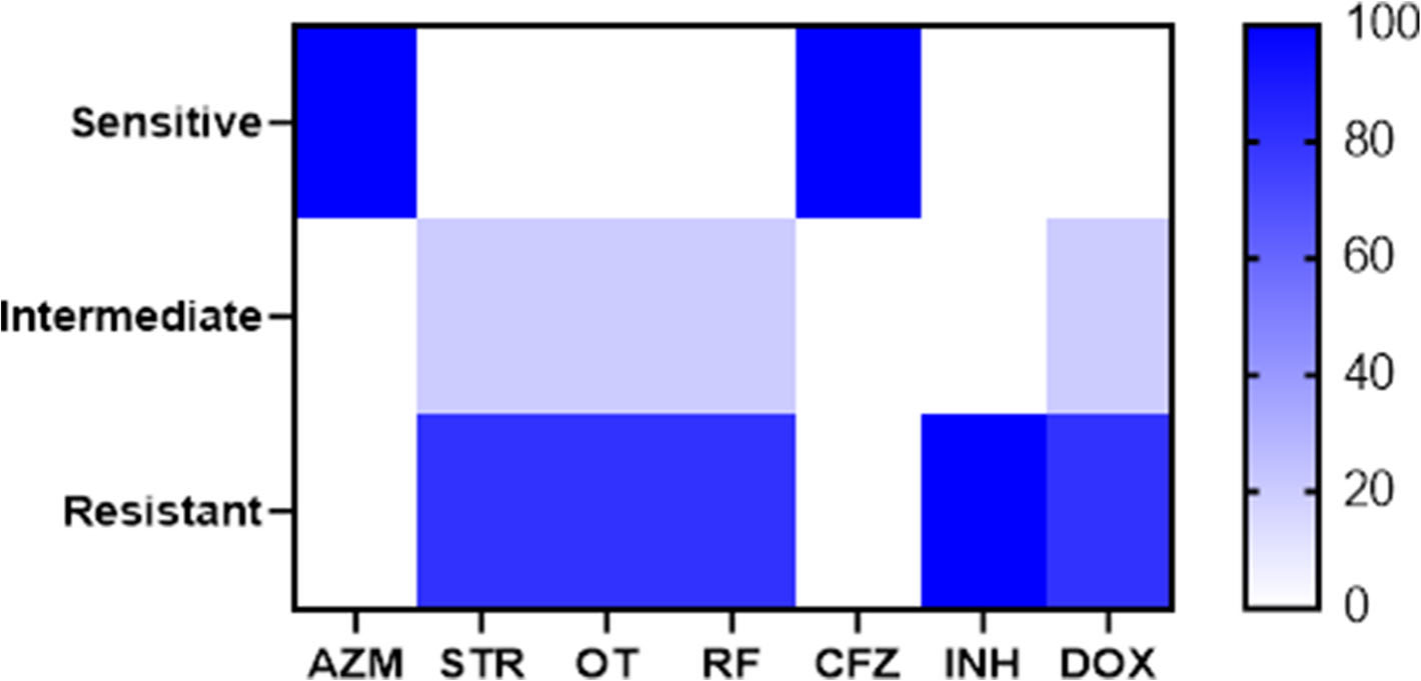
The heat map illustrates the susceptibility of the recovered *M. avium* subsp. *avium* isolates to different tested antibiotics

**Fig. 3:**
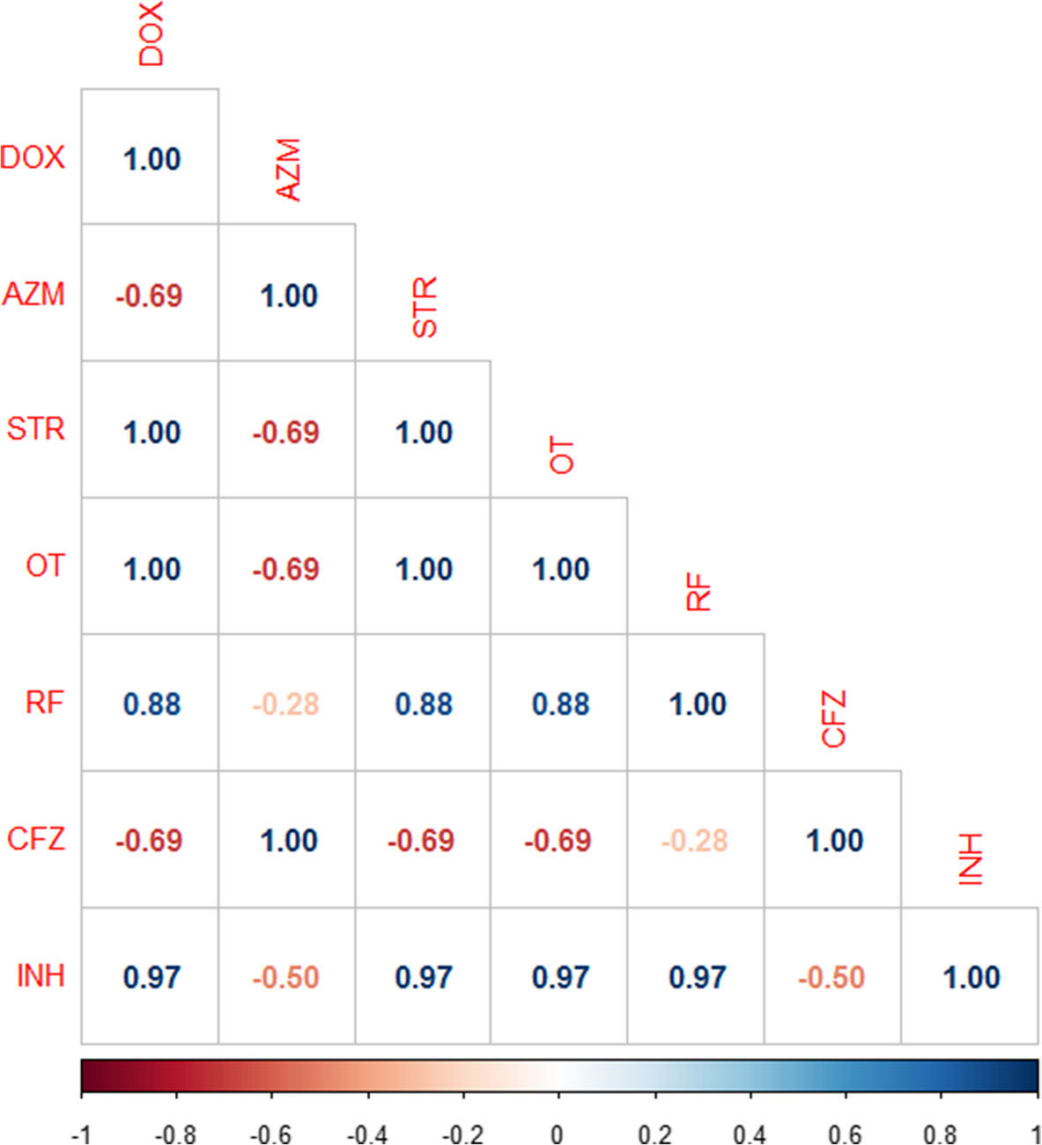
The heat map illustrates the correlation between the different tested antimicrobial agents against the recovered *M. avium* subsp. *avium* isolates. Red and blue colors refer to the negative and positive correlations, respectively

In the present study, the results of the broth microdilution method exhibited harmony with the results of the disc diffusion method. Four isolates were resistant to doxycycline, streptomycin, oxytetracycline rifampicin, and isoniazid with detectable MICs of > 8 μg/ml, > 8 μg/ml, > 8 μg/ml, > 2 μg/ml, and > 0.25 μg/ml, respectively. All the tested isolates (*n* = 5) were sensitive to azithromycin (MIC ≤2 μg/ml) and clofazimine, while only one isolate was sensitive to rifampicin. Moreover, all the tested isolates were resistant to isoniazid (MIC > 0.25 μg/ml) (Table 4 and Fig. 2). The correlation analyses were conducted among different tested antimicrobial agents. The obtained results showed strong positive correlations between (0.5–1): CFZ and AZM (r = 1); RF, STR, and OT(r = 1); DOX, STR, and OT(r = 1); OT, DOX, RF, and INH (r = 0.97); STR and OT (r = 0.97) and OT, DOX, and STR (r = 0.88) as described in Fig. 3.

**Table 4:** The results of the broth microdilution method for detection of MICs of the tested antimicrobial agents

Antimicrobial agents	Resistant			Intermediate			Sensitive		
	No	%	Interpretation μg/ml	No.	%	Interpretation μg/ml	No	%	Interpretation μg/ml
**Doxycycline**	4	80	≥8	1	20	2–4	0	0	≤ 1
**Azithromycin**	0	0	≥8	0	0	4	5	100	≤2
**Streptomycin**	4	80	≥8	1	20	4	0	0	≤2
**Oxytetracycline**	4	80	≥8	1	20	2–4	0	0	≤ 1
**Rifampicin**	4	80	≥2	0	0	–	1	20	≤ 1
**Clofazimine**	0	0	≥ 1	0	0	–	5	100	–
**Isoniazid**	5	100	≥0.25	0	0	–	0	0	≤ 0.12

### PCR-based detection of *IS901*confirmatory gene and *inh*A*, rpo*B*, rps*L*,* and *otr*B antibiotic resistance-related genes in the recovered *M. avium* subsp. *avium* isolates

**Fig. 4:**
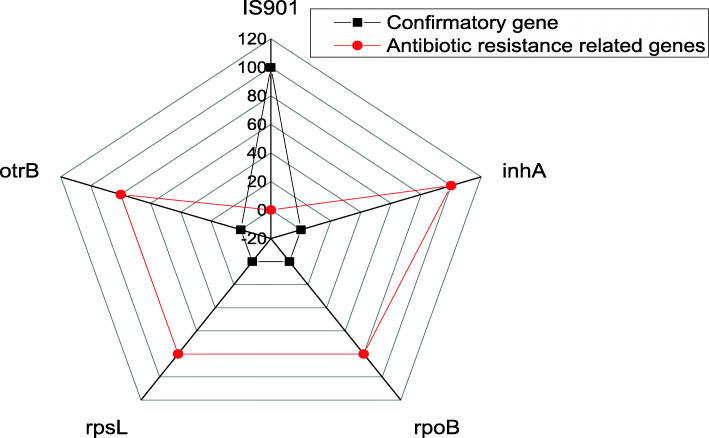
The distribution of *IS901*, *inh*A*, rpo*B*, rps*L*,* and *otr*B genes among the recovered *M. avium* subsp. *avium* isolates

In the present study, the identification of the recovered isolates is confirmed using PCR, where all the tested isolates were positive for the *IS901* confirmatory gene with a specific amplicon size of 577 bp. Moreover, PCR was used to detect the *inh*A, *rpo*B*, rps*L, and *otr*B antibiotic resistance-related genes. Our finding revealed that all the tested isolates (100%, 5/5) (four duck isolates and one goose isolate) are harbored *inh*A genes with a specific amplicon size of 810 bp. Moreover, the *rpo*B*, rps*L*,* and *otr*B resistance genes are detected in four recovered isolates (80%, 4/5) (Three of duck origin and one isolate of geese origin) with specific amplicon size of 347 bp, 592 bp, and 947 bp, respectively. The distribution of *IS901*, *inh*A*, rpo*B*, rps*L*,* and *otr*B genes among the recovered *M. avium* subsp. *avium* isolates was illustrated in Table 5 and Fig. 4. Statistically, there is no significant difference in the distribution of *IS901*, *inh*A, *rpo*B*, rps*L, and *otr*B genes in the recovered isolates (*P* > 0.05, *P* = 0.9915) as illustrated in Table 5.

**Table 5:** The distribution of *IS901* gene and antibiotic resistance-related genes associated with the retrieved isolates

Types of genes		***N***	%	Chi square ***P*** value
**Confirmatory gene**	*IS901*	5	100	0.27273 0.9915
**Antibiotic resistance-related genes**	*inh*A	5	100	
	*rpo*B	4	80	
	*rps*L	4	80	
	*otr*B	4	80

### The correlation between the phenotypic and genotypic resistance patterns of the isolated *M. avium* subsp. *avium*

**Fig. 5:**
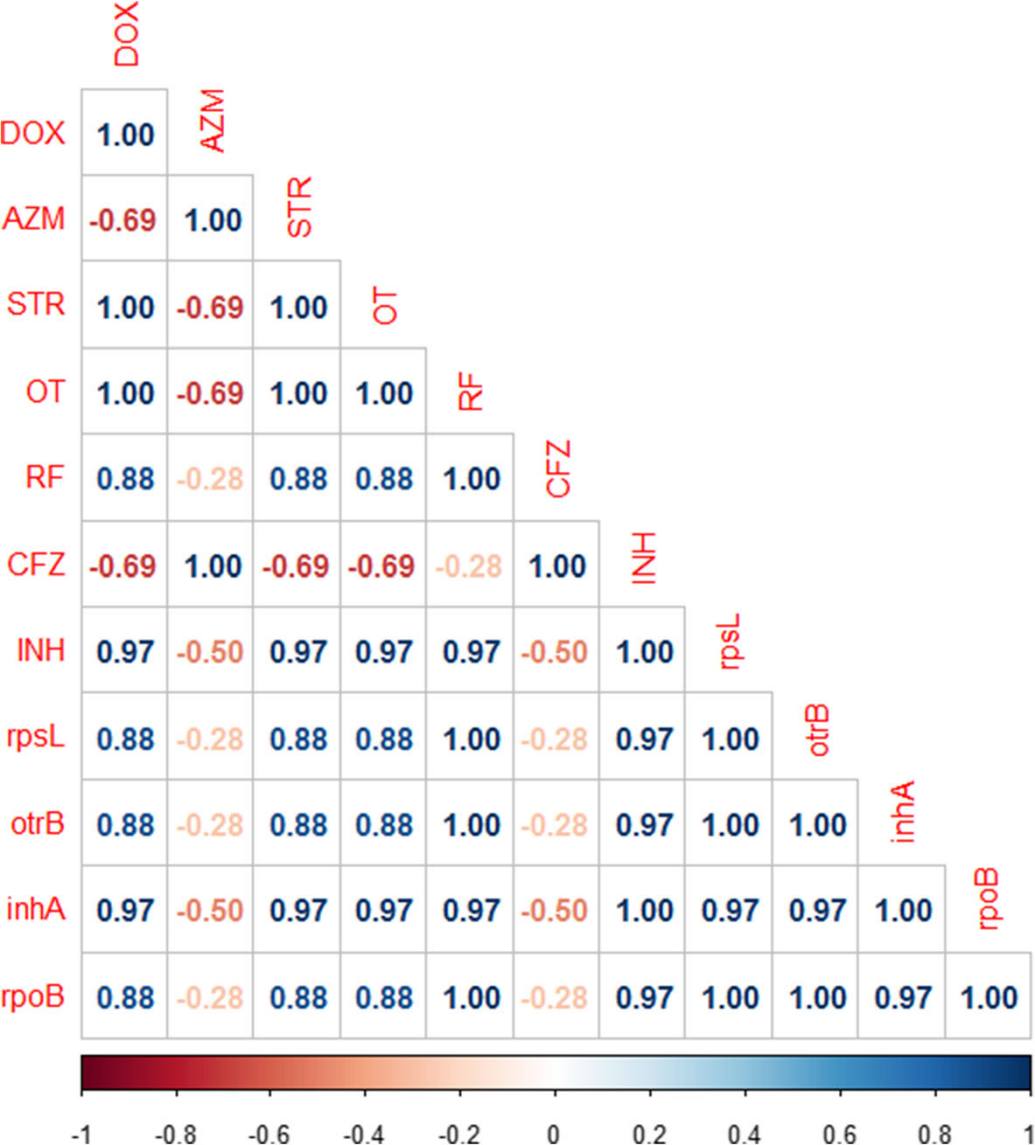
The heat map illustrates the correlation between the different tested antimicrobial agents and the antibiotic resistance-related genes in the recovered *M. avium* subsp. *avium* isolates. Red and blue colors refer to the negative and positive correlations, respectively

Regarding the correlation between the in-vitro antibiotic resistance and the genotypic resistance patterns, our finding proved that 80% (4/5) of the retrieved *M. avium* subsp. *avium* isolates (three isolates of duck origin and one isolate of geese origin) exhibited multidrug resistance patterns to isoniazid, rifampicin, streptomycin, oxytetracycline, and doxycycline, and harbored the *inh*A *rpoB, rps*L, and *otr*B genes as described in Table 6. The correlation analyses were conducted between various tested antimicrobial agents and the detected antibiotic resistance-related genes in the recovered *M. avium* subsp. *avium* isolates. Our results proved strong positive correlations (r = 0.5–1) between: *rop*B gene and RF (r = 1); *inh*A gene and INH (r = 0.97); *otr*B and OT(r = 0.88); *rps*L gene and STR (r = 0.88); *otr*B gene and DOX (r = 0.88) as illustrated in Fig. 5.

**Table 6:** Phenotypic and genotypic resistance patterns of the isolated *M. avium* subsp. *avium*

Antimicrobial Agents	Genes	Phenotypic resistance (Antibiogram)		Genotypic resistance (Antibiotic resistance-related genes)	
		No.	%	No.	%
**Isoniazid**	*inh*A	5/5	100	5/5	100
**Rifampicin**	*rpo*B	4/5	80	4/5	80
**Streptomycin**	*rps*L	4/5	80	4/5	80
**Oxytetracycline**	*otr*B	4/5	80	4/5	80

## Discussion

Regarding the phenotypic characteristics of *M. avium* subsp. *avium*, our findings proved no diversity in both morphological and biochemical characteristics among the recovered isolates and revealed a remarkable harmony between different retrieved isolates from duck and geese origins. Our results agreed with those reported by Zhu et al. [35] and Bhalla et al. [40]. The decontamination of fecal samples using 0.9% Hexadecylpryridinium Chloride Monohydrate and the antibiotic mixture is the best method to increase the recovery of *M. avium* subsp. *avium* from the fecal samples of examined birds [31]. The culture technique is considered the gold standard method used for the identification of *M. avium* subsp. *avium* as it is the most sensitive and specific method (never give false-positive results), but it requires more time [17]. On the other hand, the direct microscopical examination of acid-fast bacilli is not accurate and requires a high load of microorganisms (Approximately, 10.000 bacteria/g) to be effective. Moreover, it may give false-negative results due to its low sensitivity and specificity [41, 42].

In the present study, the frequency of *M. avium* subsp. *avium* in the examined house-reared birds agreed with that previously reported by Kindu and Dagnaw [13] who isolated *M. avium* subsp. *avium* from domestic birds in Ethiopia with a prevalence of 4.26% (12/282). Moreover, in Bangladesh, Reza et al. [43] reported that the prevalence of *M. avium* subsp. *avium* in droppings of different examined birds was 3.75% (3/80). On the other hand, in the Czech Republic, Shitaye et al. [44], and Kazda et al. [45] reported that turkeys are susceptible to *M. avium* subsp. *avium*, while ducks, geese, and water birds are resistant to avian tuberculosis. Furthermore, higher prevalence (11.1%) of *M. avium* subsp. *avium* was reported in the Czech Republic by Dvorska [46]. The prevalence of infection in birds mainly increased due to the bad hygienic conditions, overcrowding, and multi-aged birds’ populations. Moreover, some birds are highly susceptible to avian tuberculosis due to genetic factors [47, 48]. The prevalence of infection in birds mainly increased due to the bad hygienic conditions, overcrowding, and multi-aged birds’ populations. Moreover, some birds are highly susceptible to avian tuberculosis due to genetic factors [49]. The infection of birds occurs due to the ingestion of contaminated food and water with feces of infected birds, and also may occur via the respiratory route [6]. Besides, avian tuberculosis could be transmitted to the immunocompromised persons through the handling of carrier birds or via the ingestion of insufficiently cooked meat of infected birds [8].

In the current study, PCR was used to confirm the diagnosis of the recovered isolates depending on the detection of the *IS901* gene that is specific to *M. avium* subsp. *avium*. Our results emphasized the presence of the *IS901* gene in all tested isolates. Our findings agreed with those reported by Pavlik et al. [49] who detected the *IS901* gene in all tested *M. avium* subsp. *avium* strains originated from birds. *M. avium* subsp. *avium* are characterized by the existence of the specific insertion sequence *IS901* in their genome. PCR could be used as a molecular tool to detect the pathogenic variant of *M. avium* subsp. *avium* as well as the screening of elderly birds [50].

Concerning the antibiogram of the retrieved *M. avium* subsp. *avium* isolates, our findings emphasized the promising in-vitro antimicrobial activity of azithromycin and clofazimine. *M. avium complex* commonly exhibits a potential sensitivity to macrolides, especially azithromycin [51]. Moreover, Huang et al. [52] revealed that clofazimine gives optimistic results in the treatment of *M. avium*-associated infections. Higher frequencies of antimicrobial resistances were identified to doxycycline, streptomycin, oxytetracycline, rifampicin, and isoniazid using the broth microdilution method. In a previous study by Pang et al. [50], among the recovered *Mycobacterium avium* complex strains, *Mycobacterium avium* was the most resistant to the tested antimicrobials with a resistance rate of 73.68%. In the present study, most of the retrieved *M. avium* subsp. *avium* isolates were resistant to isoniazid, rifampicin, streptomycin, oxytetracycline, and doxycycline, and harbored the *inh*A, *rpo*B*, rps*L, and *otr*B genes. The multidrug resistance in *Mycobacterium* species is defined as the resistance to at least both isoniazid and rifampicin [53]. The complicated cell wall structure, the presence of mycolic acid, and the lipoidal contents are the main factors that control the intrinsic multidrug-resistance of the *M. avium complex* [15]. The resistance to rifampicin is common in the *M. avium complex* and mainly occurred due to the mutation of the *rpo*B gene that encoding for the β-subunit of the DNA-dependent RNA polymerase *M. avium complex* [54]. Moreover, the resistance to the oxytetracycline is attributed to the presence of the *otr*B gene encoding for the integral membrane protein that is responsible for the efflux of oxytetracycline from the cell [55]. Aminoglycosides exert their action by ribosomal binding near site A with subsequent inhibition of protein synthesis [56]. The Resistance to streptomycin is common in the *M. avium complex* and occurred due to mutations in the *rps*L gene encoding the ribosomal protein (S12) [57]. The *inh*A gene encodes for the enoyl acyl reductase which is involved in the mycolic acid synthesis that is responsible for the isoniazid resistance [58]. Briefly, the existence of multidrug resistance in the *M. avium complex* is attributed to several factors including 1-The intrinsic resistance due to the complex thick cell wall and the presence of mycolic acid, 2-The presence of specific antimicrobial resistance genes, and 3-Mutations that takes place in certain genes such as *rps*L and *rpo*B genes that adversely affect the activity of the antimicrobial agents [59].

### Study limitations

Gene sequencing of the antibiotic-resistance related genes of isolates (*rpo*B and *rps*L) should be carried out for the detection of mutations that adversely affect the activity of the antimicrobial agents.

## Conclusion

In conclusion, to the best of our knowledge, this is the first report that emphasized the emergence of MDR-*M.avium* subsp*. avium* in house-reared domestic birds (duck and geese) in Egypt. The emergence of MDR-*M. avium* subsp. *avium* is considered a public health threat. The combination of phenotypic and genotypic characterization is an effective epidemiological tool for the identification of *M. avium* subsp. *avium*. Emerging MDR-*M. avium* subsp. *avium* in house-reared domestic birds are commonly harbored the *IS901, inh*A*, rpo*B*, rps*L*,* and *otr*B genes. Azithromycin and clofazimine revealed a promising in-vitro antimicrobial activity against *M. avium* subsp. *avium*. The continuous monitoring of bird flocks is essential to reduce the prevalence of avian tuberculosis. The regular application of antimicrobial susceptibility testing is necessary to determine the most effective antimicrobial agent and detect the emerging MDR-strains.

## Data Availability

All the data has been included in the manuscript.

## Abbreviations

OIE: World Organization for Animal Health
CLSI: Clinical and Laboratory Standards Institute
PDR: Pan-drug resistant.
XDR: Extensive drug-resistant
MDR: Multi-drug resistant
